# Ligamentum flavum hypertrophy animal models: methodological trade-offs and future directions

**DOI:** 10.3389/fvets.2025.1703252

**Published:** 2025-11-28

**Authors:** Long Chen, Wanxia Zhang, Yu Zhang, Qin Gong

**Affiliations:** 1 Foshan Hospital of Traditional Chinese Medicine, Foshan, China; 2 The First Dongguan Affiliated Hospital of Guangdong Medical University, Dongguan, China; 3 Guangdong Provincial Second Hospital of Traditional Chinese Medicine, Guangzhou, China

**Keywords:** ligamentum flavum hypertrophy, animal models, surgical modeling, mechanical stress, collagen deposition

## Abstract

Ligamentum flavum hypertrophy (LFH) represents a key pathological factor in lumbar spinal stenosis (LSS), which is characterized by abnormal collagen deposition, reduced elastin fibers, and other degenerative changes. LFH compresses the spinal cord and nerve roots, causing symptoms like low back pain, numbness, and intermittent claudication, and may lead to central spinal stenosis, with significant consequences for quality of life. The pathophysiology of LFH involves extracellular matrix remodeling, inflammatory mediator release, and biomechanical stress, which contribute to spinal canal narrowing and nerve root compression. Although known risk factors such as age, obesity, and mechanical load have been identified, the exact mechanisms remain unclear. Well-established animal models are essential for understanding LFH and developing therapeutic strategies. This paper compares four major LFH animal models—surgical, biomechanical, chemical induction, and hybrid models—with evaluation of their clinical relevance, technical feasibility, cost-effectiveness, and limitations. Recommendations are provided for improving these models to enhance preclinical and clinical application.

## Introduction

Lumbar spinal stenosis (LSS) features narrowing of the spinal canal along with compression of the spinal cord and nerve roots. Ligamentum flavum hypertrophy (LFH) frequently occurs in LSS and contributes significantly to disease progression (1, 2). LFH shows higher prevalence in middle-aged and elderly populations, and its incidence is increasing with global aging (3). The ligamentum flavum (LF) is mainly composed of elastin and collagen fibers (4). LFH arises from excessive collagen deposition combined with loss of elastin (5–7). This thickening exerts mechanical pressure onto surrounding spinal structures, leading to symptoms including low back pain, numbness, and intermittent claudication. In advanced cases, LFH may evolve into central spinal stenosis, frequently accompanied by significant neurological deficits (3).

The pathogenesis of LFH is multifactorial, involving extracellular matrix remodeling, inflammation, and biomechanical stress (8–11). These factors drive progressive thickening of LFH and spinal canal narrowing, ultimately compressing nerve roots and causing neurological impairment. While age, mechanical stress, obesity, and metabolic abnormalities correlate with LFH development, the precise pathogenic mechanisms require further elucidation, presenting therapeutic challenges (12–15).

Animal models play a vital role in investigating LFH pathogenesis and therapy evaluation, enabling exploration of disease mechanisms, treatment testing, and preclinical assessment of new therapies. However, model development encounters obstacles in pathological fidelity, feasibility, and clinical translatability. This review summarizes current models—surgical, biomechanical, chemical induction, and hybrid—with evaluation of their clinical relevance, complexity, cost-effectiveness, and limitations, along with suggested improvements and projected future research directions for LFH.

## Model classification and critical evaluation

### Traditional surgical models: the dilemma between standardization and over-simplification of pathological simulation

#### Methodology

In traditional surgical animal models, LFH is induced by destabilizing the lumbar spine. For instance, Hayashi et al. (16) conducted L2–3 and L4–5 posterolateral spinal fusion in experimental rabbits to induce LFH, which led to collagen fiber deposition and increased TGF-β1 expression. Similarly, Wang et al. (11) increased local mechanical stress by resecting spinous processes, paraspinal muscles, or facet joints. In rat models, this approach has been widely used, with studies showing that resecting the L5–L6 spinous processes and paraspinal muscles significantly increases the collagen fiber ratio in the ligamentum flavum (LF). These techniques entail surgical disruption of spinal stability to trigger compensatory LFH.

#### Advantages

This modeling approach offers distinct advantages in pathological reproducibility and cost-effectiveness. Rat models are relatively inexpensive and easy to implement.

#### Limitations

Traditional surgical models oversimplify LFH pathology, without replicating its complex mechanisms. They induce biological responses that may not reflect true disease changes and lack quantification of mechanical stress, thus affecting reliability. Variability in animal activity, influenced by the environment, increases model failure risk. Additionally, the absence of continuous monitoring limits accurate simulation of physiological load responses.

#### Future directions

Future studies need to improve mechanical stress quantification and integrate real-time monitoring of dynamic loads for better model reliability and biological relevance.

### Biomechanical models: chronic stress simulation and the limitations in inflammatory response

#### Methodology

A novel LFH model was developed by Zheng et al. (17), where mice were induced to stand on their hind legs in response to water avoidance. Over time, the cross-sectional area of the LF increased, and after about 10 weeks, collagen and elastic fibers accumulated. Similarly, Saito et al. (18) employed a mechanical stress device that applied continuous flexion and extension to the mouse spine to simulate mechanical loading. After 12 weeks, this model showed a significant increase in the cross-sectional area and thickness of the lumbar LF.

#### Advantages

Biomechanical models provide a straightforward and economical approach, well-suited to large-scale investigations.

#### Limitations

Such models introduce variability, particularly the bipedal standing model, compromising reproducibility. Although inducing LFH via mechanical stress, they do not reproduce key human features, including macrophage infiltration and TGF-β1 upregulation. Additionally, visualizing the LF in mice is challenging due to its thinness, hindering imaging and histopathology. Tissue collection for techniques like Western blot (WB) or PCR is also difficult, restricting molecular analysis.

#### Future directions

Subsequent research should improve mechanical stress quantification and establish real-time monitoring systems to more accurately simulate human LFH. Employing rats, which are easier to obtain specimens from, instead of mice could enhance experimental feasibility. New models should incorporate inflammatory elements, like macrophage infiltration and TGF-β1 upregulation.

### Chemical induction models: precision of molecular targeting and the lack of biomechanical context

#### Methodology

Zhou et al. (19) employed a chemical induction model by implanting Lysophosphatidic acid (LPA)-loaded gelatin sponges into the epidural space following partial resection of the spinous processes, supraspinous ligaments, and interspinous ligaments in rats. This procedure successfully activated the LPAR1-Akt signaling pathway, promoting cell proliferation and inhibiting apoptosis in ligamentum flavum (LF) cells, thereby causing ligament hypertrophy. This model allowed for precise molecular targeting to study the fibrotic mechanisms behind LFH.

#### Advantages

The chemical induction model targets specific molecular pathways, providing a more accurate tool for studying LFH mechanisms. It isolates fibrotic processes without mechanical stress interference, facilitating focused molecular investigations.

#### Limitations

The chemical induction model targets specific molecular pathways but fails to replicate the biomechanical environment of LFH, restricting its clinical relevance. It is better suited for studying fibrosis but cannot simulate the disease’s full progression. Additionally, LPA application increases costs and limits widespread adoption.

#### Future directions

Future studies should integrate chemical induction with biomechanical loading to simulate both molecular and mechanical factors of LFH. Exploring cost-effective inducers and improving LF visualization in small animals will increase model utility.

### Hybrid models: the trade-off between accurate simulation and high costs

#### Methodology

Chen et al. (20) an innovative hybrid model was developed, integrating both surgical interventions and dynamic mechanical loading. In this model, the L5-L6 spinous processes, transverse processes, and supraspinous ligament were resected, followed by dynamic load training using a treadmill. After 7 weeks, an increase in LF thickness was observed in rats, with hallmark changes similar to those seen in human LFH, including increased expression of COL1 and TGF-β1.

#### Advantages

The hybrid approach demonstrates clear advantages compared to conventional techniques, primarily through a reduced modeling timeline and improved biological relevance. Its dynamic loading component effectively reproduces human activity patterns, which enhances biological fidelity and more accurately represents physiological conditions. Consequently, this model emerges as a valuable tool for LFH research, enabling the investigation of disease mechanisms within a context that better mirrors biology.

#### Limitations

Although biologically accurate, this hybrid model presents certain constraints. The involved surgical procedure demands specialized equipment, elevating expenses and technical challenges. Postsurgical trauma can impact rodent survival rates as well. Whereas the model displays precise short-term biological responses, its long-term outcomes and reproducibility require additional verification. Dependence on sophisticated methodologies restricts its broad application in standard research settings.

#### Future directions

Subsequent research should simplify surgical procedures to reduce complexity and costs while maintaining biological accuracy. Long-term effects and reproducibility must be more thoroughly validated for reliable extended studies. Minimizing surgical trauma and improving recovery will enhance model feasibility. The goal is to create a cost-effective hybrid model with high biological relevance to human LFH.

## Discussion

Available animal models advance our understanding of LFH pathophysiology and treatment options. Yet all exhibit limitations in replicating human disease complexity. Surgical and biomechanical models replicate pathology but overlook molecular details. Chemical models target molecular pathways effectively but lack biomechanical context, reducing clinical relevance. Hybrid models improve biological accuracy but require complex, costly procedures. Subsequent studies must balance precision with practicality in model design.

Subsequent studies should combine biomechanical loading and molecular induction to replicate LFH’s multifactorial pathogenesis. Real-time monitoring via wearable devices and dynamic imaging will better track mechanical stress and fibrosis progression, enhancing model relevance. Advances in 3D bioprinting may enable humanized LF organoids, creating new platforms for cellular research and drug testing. Multi-omics approaches like single-cell RNA sequencing and spatial transcriptomics can uncover cellular heterogeneity and molecular networks, elucidating LFH mechanisms. Integrating these innovative methods will yield models with improved clinical translation potential, supporting early diagnosis and personalized LFH treatment.

## Conclusion

An ideal LFH animal model must balance scientific accuracy with practical constraints. Emerging technologies, including 3D bioprinting and AI-driven platforms, combined with collaborative research initiatives, present opportunities to address current model limitations. Such advances could enable more robust and clinically relevant models that strengthen both scientific rigor and therapeutic applications.

## Data Availability

The original contributions presented in the study are included in the article/supplementary material, further inquiries can be directed to the corresponding authors.
